# Codamozza‐Fluker: The Compelling Case of a Flukeless Fin Whale Traveling Throughout the Mediterranean Sea and the Need for Basin‐Wide Conservation Efforts

**DOI:** 10.1002/ece3.71313

**Published:** 2025-05-21

**Authors:** Maddalena Jahoda, Margherita Zanardelli, Frank Dhermain, Jessica Alessi, Filippo Armonio, Marco Ballardini, Alain Barcelo, Giulia Calogero, Elena Fontanesi, Alexandros Frantzis, Maria Assunta Menniti, Clara Monaco, Céline Obadia, Giuliana Pellegrino, Alessandra Raffa, Céline Tardy, Alessandro Verga, Biagio Violi, Simone Panigada

**Affiliations:** ^1^ Tethys Research Institute Milano Italy; ^2^ MIRACETI Martigues France; ^3^ PELAGIS Réseau National D'échouage La Rochelle France; ^4^ MeRiS—Mediterraneo Ricerca e Sviluppo APS Favara (AG) Italy; ^5^ Blue Conservancy ETS Brancaleone (RC) Italy; ^6^ Costa Balenae Imperia Italy; ^7^ Parc National de Port‐Cros Hyeres Cedex France; ^8^ Menkab: Il Respiro del Mare Savona Italy; ^9^ Delfini del Ponente APS Imperia Italy; ^10^ Pelagos Cetacean Research Institute Vouliagmeni Greece; ^11^ CESRAM, Centro Studi e Ricerca Ambiente Marino Guardavalle (CZ) Italy; ^12^ Marecamp Association Acicastello (CT) Italy; ^13^ Golfo Paradiso Whale Watching Camogli (GE) Italy

**Keywords:** anthropogenic impacts, *Balaenoptera physalus*, collisions, ecosystem‐based conservation, fin whale, flukeless whale, Mediterranean Sea, migration

## Abstract

Human‐caused injuries can have severe consequences for cetaceans, both for the well‐being of individuals and the effects at the population level. Here, we report the first case of a live fin whale in the Mediterranean Sea with completely amputated flukes, a female who suffered two separated incidents: the first one before 1996, when she lost her left lobe, and the second one, in 2019, leading to the severing of the whole tail and finally to presumed death. “Codamozza‐Fluker” was regularly sighted during summer in the North‐Western Mediterranean for 24 years. Entanglement and collision were both hypothesized as potential causes of her injuries. Without flukes, she traveled at least 7000 km across the Mediterranean, despite severe emaciation. Sighting records allowed tracking her from September 2019 to July 2020, providing rare evidence of large‐scale movements within the Mediterranean over almost a year. Her journey, which extended as far as Syria, provides the first evidence of a fin whale from the Ligurian Sea being resighted in the Eastern Basin. This introduces a new perspective on this endangered subpopulation's movement patterns, which are crucial for effective conservation measures. The case gained widespread media attention, representing a dramatic example of usually unnoticed injuries or deaths, and contributed to raising public awareness, essential for driving focused conservation efforts. Her latest accident most likely occurred within the Pelagos Sanctuary for marine mammals, highlighting the need for more effective local protection measures, and underscoring the importance of complementing area‐based conservation efforts with an ecosystem‐based approach.

## Graphical abstract

The authors report the first case of a live fin whale in the Mediterranean Sea with completely amputated flukes, a female which suffered two separated incidents with entanglement and collision both being hypothesized as causes. Sightings after the complete loss of her flukes enabled tracking her movements from September 2019 to July 2020, prior to her presumed death, providing rare evidence of large‐scale movements—at least 7000 km—within a span of nearly a year. This also represents the first documented case of a fin whale from the Ligurian Sea being re‐sighted in the Eastern Mediterranean Basin. Her latest accident, which attracted widespread media attention, likely occurred within the Pelagos Sanctuary for Marine Mammals, underscoring the need for more effective protection measures.

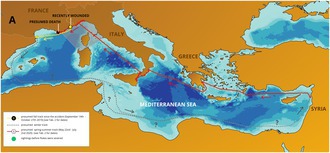

## 1 Introduction

Worldwide, there are several documented cases of large whales with body parts amputated due to anthropogenic causes, with the most dramatic records involving individuals that have lost both flukes. The tail is the main source of propulsion for cetaceans (Huggenberger et al. 2018); nevertheless, some individuals survive despite this significant impairment, at least temporarily, even traveling long distances. However, such injuries ultimately lead to a slow death, likely accompanied by significant suffering. Moreover, the loss of those individuals can cause a negative impact on the viability of a population.

Based on records we were able to collect, the loss of one or both flukes in whales has been recorded since 1956, but only since the 1970s on a more regular basis (Table 1). Aside from 26 cases in which the animals died due to their wounds, we recorded 44 cases of large cetaceans with amputations but still living, 23 (52.3%) of which account for completely flukeless individuals.

**TABLE 1:** Flukeless whales due to presumed anthropogenic causes, worldwide. Only *Mysticetes* and sperm whales were considered, and only cases where the whole or a substantial part of the tail was missing.

No.	Alive whales									
	Year	Month	Species	Location	Ocean/sea	Injury type	Gender	Age class	Notes	Reference
1	1956	June	*Megaptera novaeangliae*	Off S. Francisco, CA, USA	Pacific	One fluke missing	F	Adult		Gilmore (1959)
2	1958	February	*Eschrichtius robustus*	*S. Diego* Bay, CA, USA	Pacific	Flukeless			Possibly the same whale sighted in May off Kodiak Island, AK, USA	Gilmore (1959)
3	1958		*Megaptera novaeangliae*	S. Francisco Bay, CA, USA	Pacific	One fluke missing				Gilmore (1959)
4	1958		*Balaenoptera musculus*	S. Francisco Bay, CA, USA	Pacific	One fluke missing				Gilmore (1959)
5	1976	(winter)	*Eschrichtius robustus*	S. Ignacio Lagoon, Baja California, Mexico	Pacific	Flukeless				Urbàn et al. (2004)
6	1976	February	*Eschrichtius robustus*	Fry's Harbor, Santa Cruz island, CA, USA	Pacific	Flukeless				Patten et al. (1980)
7	1978	February	*Eschrichtius robustus*	Anacapa Island, CA, USA	Pacific	Flukeless			Possibly the same individual sighted in March south of Point Fermin, Palos Verdes Peninsula, and in April near Catalina Island (CA, USA)	Patten et al. (1980)
8	1979	March	*Eschrichtius robustus*	Ensenada, Baja California, Mexico	Pacific	Flukeless				Patten et al. (1980)
9	1980	March	*Eschrichtius robustus*	Boca de Soledad, Magdalena Bay, Baja California, Mexico	Pacific	Flukeless				Patten et al. (1980)
10	1982	February, March	*Eschrichtius robustus*	S. Ignacio Lagoon, Magdalena Bay, Baja California, Mexico	Pacific	Flukeless	F	Adult with calf		Urbàn et al. (2004)
11	1984	March	*Eschrichtius robustus*	Ensenada, Baja California, Mexico	Pacific	Flukeless				Janiger (pers. comm.)
12	1987	August	*Eubalaena glacialis*	Browns Bank, Canada	Atlantic	one fluke missing				Knowlton and Kraus (2001)
13	1989	Winter	*Eschrichtius robustus*	S. Ignacio Lagoon, Baja California, Mexico	Pacific	Flukeless				Urbàn et al. (2004)
14	1992	Winter	*Eschrichtius robustus*	S. Ignacio Lagoon, Baja California, Mexico	Pacific	Flukeless				Urbàn et al. (2004)
15	1994	June	*Balaenoptera physalus*	Pelagos Sanctuary, Italy, France	Mediterranean Sea	One fluke missing		Adult		Tethys Research Institute data
16	1997	February	*Eschrichtius robustus*	S. Ignacio Lagoon, Baja California, Mexico	Pacific	Flukeless		Adult	Sighted again twice in February–March	Urbàn et al. (2004)
17	1998	January	*Eubalaena glacialis*	Georgia, USA	Atlantic	One fluke missing				Jensen and Silber (2003)
18	1998	June	*Balaenoptera physalus*	Pelagos Sanctuary, Italy, France	Mediterranean Sea	One fluke missing		Adult		Tethys Research Institute data
19	2001	January	*Eschrichtius robustus*	Off Montana de Oro State Park, CA, USA	Pacific	Flukeless		Calf		Jensen and Silber (2003)
20	2002	December	*Megaptera novaeangliae*	Bahia de Banderas, Nayarit, Mexico	Pacific	Flukeless				Lugo and Rodriguez (2003)
21	2005	March	*Eubalaena glacialis*	Cumberland Island GA, USA	Atlantic	Partially severed left fluke			Resighted in April 2005	Glass et al. (2008)
22	2005	June	*Balaenoptera physalus*	Pelagos Sanctuary, Italy, France	Mediterranean Sea	One fluke missing		Adult		Ballardini et al. (2006)
23	2006	July	*Physeter macrocephalus*	Off SW Crete, Hellenic Trench, Greece	Mediterranean Sea	Largest part of the left fluke missing		Immature/adult	First observed in 2000, then in 2006 and 2007.	Pelagos Cetacean Research Institute, East Med sperm whale photo‐identification database
24	2006	August	*Eubalaena glacialis*	Great South Channel, MA, USA	Atlantic	Nearly severed flukes		Adult		Glass et al. (2008)
25	2008	August	*Megaptera novaeangliae*	Monterey, CA, USA	Pacific	Flukeless				Carretta et al. (2014)
26	2009	February	*Eubalaena glacialis*	Ponta Vedra, FL, USA	Atlantic	One fluke missing	F	Newborn		Sharp et al. (2019)
27	2009	August	*Physeter macrocephalus*	Pelagos Sanctuary, Italy, France	Mediterranean Sea	Over half of right fluke missing		Adult		Tethys Research Institute data
28	2015	March	*Eschrichtius robustus*	Dana Point, CA, USA	Pacific	Flukeless			Also sighted off Point Vicente, spanning a 3 day period. NOAA officials presumed it is the same found dead in Mendocino the same year, although no confirmed match.	Janiger (pers. comm.)
29	2016	March	*Megaptera novaeangliae*	Virginia Beach, MA	Atlantic	One fluke missing				Henry et al. (2020)
30	2016	July	*Megaptera novaeangliae*	Kaikoura, NZ	Pacific	Flukeless		Juvenile		https://www.bbc.com/news/world‐asia‐35818690
31	2016	July	*Megaptera novaeangliae*	2.4 nm SE of Chatham, MA, USA	Atlantic	Flukes compromised and deteriorating				Henry et al. (2020)
32	2017	August–September	*Balaenoptera physalus*	St. Lawrence river estuary, Quebec, Canada	Atlantic	Part of flukes missing				Timothée Perrero (pers. comm.)
33	2018	March	*Eschrichtius robustus*	Oceanside, *S. Diego* County, CA, USA	Pacific	Flukeless			Likely the same individual seen in Newport Beach in February	Janiger (pers. comm.)
34	2018	April	*Eschrichtius robustus*	Malibu, CA, USA	Pacific	Flukeless			Likely the same spotted in the Port of Los Angeles	Janiger (pers. comm.)
35	2018	August	*Megaptera novaeangliae*	Nuqui, Tribugà Gulf, Chocò, Colombia	Pacific	Flukeless		Juvenile		Natalia Botero Acosta, Fundación Macuáticos Colombia (pers. comm.)
36	2018	August	*Megaptera novaeangliae*	Humboldt, CA, USA	Pacific	Flukes compromised		Juvenile		Carretta et al. (2021)
37	2019	September	*Balaenoptera physalus*	Cap Ferrat, France	Mediterranean Sea	Flukeless	F	Adult		Case object of this article
38	2019	October	*Megaptera novaeangliae*	Los Angeles, CA, USA	Pacific	Damaged or missing right fluke				Carretta et al. (2021)
39	2019	November	*Megaptera novaeangliae*	*S. Diego* , CA, USA	Pacific	Progressing vs. amputation of tail				Carretta et al. (2021)
40	2020	August	*Balaenoptera physalus*	Off Sanremo/Imperia, Pelagos Sanctuary, Italy	Mediterranean Sea	One fluke completely, one partially missing		Adult		Tethys Research Institute—Delfini del Ponente data
41	2021	September	*Megaptera novaeangliae*	1.3 nm off Sea Girt, NJ, USA	Atlantic	Flukeless				Henry et al. (2022)
42	2021	October	*Physeter macrocephalus*	Off Genova, Pelagos Sanctuary, Italy	Mediterranean Sea	Damaged right fluke		Juvenile	Resighted several times in the Pelagos Sanctuary between Genoa and San Remo and in the waters off Ischia (Tyrrhenian Sea)	Violi et al. (2023)
43	2023	March	*Eschrichtius robustus*	Newport beach, CA, USA	Pacific	Flukeless			Sighted also in Point Dume, one day apart	Janiger (pers. comm.)
44	2024	July	*Megaptera novaeangliae*	San Juan Islands, WA, USA (S of Lopez Island)	Pacific	Flukeless		Adult		Whale Museum of Friday Harbor, WA, USA https://www.facebook.com/watch/?v=506118835285966

	Dead whales									
45	1972	January	*Eubalaena glacialis*	Texas, USA	Atlantic	Flukeless		Calf		Jensen and Silber (2003)
46	1975	January	*Eschrichtius robustus*	Off Pt. Loma, CA, USA	Pacific	Flukeless				Jensen and Silber (2003)
47	1979	March	*Eubalaena glacialis*	Long Island, NY, USA	Atlantic	Flukeless		Juvenile		Laist et al. (2001)
48	1983	February	*Eubalaena glacialis*	Island Beach, NJ, USA	Atlantic	Flukeless	M	Adult		Laist et al. (2001)
49	1993	August	*Eubalaena australis*	Between Long Beach and Kopie Alleen, Cape Town, South Africa	Atlantic/Indian	Flukeless		Calf		Laist et al. (2001)
50	1993	October	*Eubalaena australis*	Lekkerwater, De Hoop, South Africa	Atlantic/Indian	Flukeless	F	Calf		Laist et al. (2001)
51	1994	March	*Balaenoptera physalus*	Cape Henry, Cheasapeake Bay, VA, USA	Atlantic	Flukeless	F	Adult		Jensen and Silber (2003)
52	1997	February	*Balaenoptera physalus*	Los Angeles Harbor, CA, USA	Pacific	Flukeless		Calf		Jensen and Silber (2003)
53	1998	July	*Eubalaena australis*	Die Dam, Quoin Point, South Africa	Atlantic/Indian	Flukeless	F	Calf		Laist et al. (2001)
54	2004	November	*Eubalaena glacialis*	Ocean Sands, Corolla, NC	Atlantic	Left fluke severed	F	Adult		Glass et al. (2008)
55	2005	June	*Balaenoptera edeni*	Between Great and Little Barrier Islands, New Zealand	Pacific	Flukeless	F			IWC Global Ship Strikes Database
56	2006	January	*Eubalaena glacialis*	Mayport, FL, USA	Atlantic	Flukeless	M	Calf		Sharp et al. (2019)
57	2008	February	*Eubalaena glacialis*	Jacksonville, FL, USA	Atlantic	Flukeless	M	Newborn		Sharp et al. (2019)
58	2009	August	*Megaptera novaeangliae*	Humboldt, CA, USA	Pacific	Flukeless				Carretta et al. (2014)
59	2011	April	*Physeter macrocephalus*	St. Cruz, Tenerife, Canary Islands, Spain	Atlantic	Flukeless	M			IWC Global Ship Strikes Database
60	2012	April	*Balaenoptera musculus*	Mirissa, Sri Lanka	Indian	almost severed tail	M			De Vos et al. (2013)
61	2014	May	*Megaptera novaeangliae*	Los Angeles, CA, USA	Pacific	Flukeless				Carretta et al. (2016)
62	2016	January	*Balaenoptera acutorostrata*	Ross Beach, Cromarty, UK	Atlantic	Flukeless		Calf		IWC Global Ship Strikes Database
63	2016	September	*Megaptera novaeangliae*	Tillamook, OR, USA	Pacific	Flukeless				Carretta et al. (2021)
64	2017	October	*Eubalaena glacialis*	Nashawena Island, Cape Cod, MA, USA	Atlantic	Flukeless	M	Calf/juvenile		Sharp et al. (2019)
65	2018	March	*Eschrichtius robustus*	Marin, CA, USA	Pacific	flukes and distal penduncle missing	F	Juvenile		Carretta et al. (2021)
66	2018	July	*Megaptera novaeangliae*	Napeague, Long Island, NY, USA	Atlantic	flukes partially severed				Henry et al. (2022)
67	2019	April	*Eschrichtius robustus*	Contracosta CA, USA	Pacific	Entire peduncle and flukes missing	F	Adult		Carretta et al. (2021)
68	2019	June	*Megaptera novaeangliae*	Cape Town harbor, South Africa	Atlantic/Indian	Flukeless		Juvenile		IWC Global Ship Strikes Database
69	2019	October	*Physeter macrocephalus*	Faroe Islands, Denmark	Atlantic	Flukeless		Calf/juvenile		IWC Global Ship Strikes Database
70	2020	December	*Balaenoptera physalus*	Saronikos Gulf, Greece	Mediterranean Sea	Flukeless		Juvenile/immature		Pelagos Cetacean Research Institute, National Stranding Database

From our records, free‐ranging, living, large whales (*Mysticetes* or sperm whales), partially or completely flukeless, belong to six species. The most frequent were gray whales ( *Eschrichtius robustus* ) (*N* = 16; 36.4%) and humpback whales ( *Megaptera novaeangliae* ) (*N* = 13; 29.5%). The remaining were six fin whales ( *Balaenoptera physalus* ), five North Atlantic right whales (*Eubalena glacialis*), one blue whale ( *Balaenoptera musculus* ), and three sperm whales ( *Physeter macrocephalus* ). Additionally, 26 whales were found dead with severed or partially severed flukes: 10 right whales (gen. *Eubalaena*), 5 humpback whales, 3 gray whales, 3 fin whales, 1 blue whale, 2 sperm whales, 1 minke whale ( *Balaenoptera acutorostrata* ), and 1 Bryde's whale ( *Balaenoptera edeni* ).

To our knowledge, no completely flukeless fin whale had ever been sighted alive; only three dead cases are on record: one with propeller marks (VA, USA), another one brought on the bow of a freighter (CA, USA), and one in Greece. In the Mediterranean Sea, no other cases of living tail‐less fin (or other) whales have been reported so far, while six more individuals, aside from the case in this study, showed the amputation of one tail lobe. All of them, but one, were sighted in the International Sanctuary for the Protection of Mediterranean Marine Mammals (hereafter “Pelagos Sanctuary”), NW Mediterranean Sea:

1. In June 1994, an adult fin whale was observed in the Ligurian Sea with a large part of its left fluke missing (Photos and data by Jonathan Gordon/IFAW to Tethys Research Institute, hereafter “TRI”).
2. In June 1998, an adult fin whale with its right lobe missing in the Ligurian Sea (TRI data).
3. A sperm whale named “Palaiospot” was first observed in 1998 in SW Crete, Greece. Its flukes were never observed until July 2006, when an underwater video showed that most of the left fluke was missing. The whale was resighted in 2007 (Pelagos Cetacean Research Institute, East Med sperm whale photo‐identification database).
4. In June 2005, a fin whale missing almost the entire right part of its flukes was observed in the Ligurian Sea (Ballardini et al. 2006).
5. In 2009, a sperm whale named “Half,” with a large part of its right fluke missing, in the Ligurian Sea (TRI data).
6. In August 2020 an adult fin whale, named “Mezzacoda” (Half‐tail), was close to the coast off Sanremo, Italy, in the Ligurian Sea. It had the right lobe of its flukes amputated and only a stripe of the other one remaining, and a deep V‐shaped scar on the peduncle, likely pointing to a collision with a vessel (TRI data).
7. In October 2021, a juvenile sperm whale named “Atlante” with clear signs of an interaction with a propeller that had removed up to 30% of its flukes (Violi et al. 2023).

These cases are the most dramatic ones, likely representing just the tip of the iceberg of the effects of heavy anthropogenic impacts on Mediterranean fin and sperm whales. Regarding fin whales, their small, isolated subpopulation in the Mediterranean Sea numbers no more than 1700 estimated mature individuals, with a decreasing trend, and has been recently uplisted from “Vulnerable” to “Endangered” in the IUCN Red List of Threatened Species (Panigada et al. 2021).

In this article, we describe a further case, the first of a free‐ranging fin whale in the Mediterranean Sea, a female with completely amputated flukes, and her journey across the Basin.

This whale suffered two distinct and temporally separated accidents: one before August 1996 when she was first sighted with her left fluke already almost entirely missing. Since then, she was regularly observed in the Pelagos Sanctuary and was named “Codamozza” (Chopped tail) in Italy and “Fluker” in France. The second accident occurred in late summer 2019, leading to the severing of the whole tail and finally to presumed death. Once fluke‐less, her long‐range movements across the entire basin could be tracked through sightings for over 9 months.

In addition to shedding some light on the still poorly understood seasonal movements of Mediterranean fin whales, the impressive case of a fluke‐less whale swimming across the entire basin also had an important impact on public awareness, drawing attention to the need to act for the conservation of local cetaceans.

## 2 Methods

Records of Codamozza‐Fluker (hereafter “Codamozza”) were opportunistically collected by different teams of researchers, whale watching operators, fishermen, and recreational boaters (Table 2). All sightings included date, photo‐identification pictures, and position recorded by GPS whenever available. Sighting points were mapped and analyzed by QGIS.

**TABLE 2:** Sightings of *Codamozza* before and after the severing of the whole tail. Estimated points (est.) were taken into account for the statistical analyses only if an accurate description of the location was available.

	Date	Lat	Long	Area	Source	Min dist. from coast (m)	Depth (m)
		After the first accident (only part of flukes missing)					
	1996, August 14th	43.5985 N	8.1415 E	Pelagos Sanctuary	TRI	32,000	2468
	2001, September 8th	43.7419 N	8.2153 E	Pelagos Sanctuary	BLUWEST	20,200	2211
	2004, May 27th	43.7228 N	8.0981 E	Pelagos Sanctuary	BLUWEST	17,800	1787
	2004, August 27th	43.5825 N	8.1283 E	Pelagos Sanctuary	TRI	32,600	2474
	2004, August 27th	43.5744 N	8.155 E	Pelagos Sanctuary	BLUWEST	34,800	2484
	2005, August 6th	43.5022 N	8.1919 E	Pelagos Sanctuary	BLUWEST/UNIGE	42,700	2508
	2005, August 18th	43.5017 N	7.3697 E	Pelagos Sanctuary	TRI	18,900	1877
	2005, August 25th	43.2818 N	7.2094 E	Pelagos Sanctuary	TRI	30,500	2243
	2006, June 21st	42.9351 N	7.7155 E	Pelagos Sanctuary	WWF FRANCE	82,400	2676
	2006, October 6th	43.0739 N	7.4156 E	Pelagos Sanctuary	WWF FRANCE	57,600	2620
	2009, June 17th	42.8577 N	5.7176 E	West of Pelagos Sanctuary	WWF FRANCE	23,200	2262
	2010 July 11th	42.900 N	5.8300 E	West of Pelagos Sanctuary	DHERMAIN/DÉCOUVERTE DU VIVANT	16,500	2200
	2013, June 16th	43.3372 N	8.2179 E	Pelagos Sanctuary	WWF FRANCE	60,800	2560
	2013, September 19th	43.6268 N	7.3104 E	Pelagos Sanctuary	SOS GRAND BLEU	5450	1102
	2018, July 9th	43.75 N	8.25 E	Pelagos Sanctuary	GOLFO PARADISO WHALE WATCHING	21,200	1980
	2019, August 18th	42.7098 N	6.2197 E	West of Pelagos Sanctuary	VERTICAL HORIZON	35,600	2522

Nr in Figure 1		After the second accident (flukeless)					
	2019, September 14th	_	_	St. Jean Cap Ferrat, Nice, France	FISHERMEN	_	_
**1**	2019, October 5th	43.3809 N	7.3548 E	St. Jean Cap Ferrat, Nice, France	SOS GRAND BLEU	25,470	1997
**2**	2019, October 13th	43.0237 N	6.4304 E	Ile du Levant, France	JOHANN CERISIER, PORT‐CROS NATIONAL PARC	447	21
**3**	2019, October 26th	42.4510 N	3.2333 E	French/Spanish border	SAMUEL ELGRISHI, ROUSSILLON FISHING	4740	94
**4**	2019, October 27th	42.5300 N	3.4300 E	Lacaze Duthiers canyon, Cap Béar	OLIVIER LARREY DÉCOUVERTE DU VIVANT	23,200	500
**5**	2020, May 22nd	35.3666 N (est.)	35.7149 E (est.)	Jablah, Syria	FISHERMEN	_	
**6**	2020, June 5th	37.1167 N	22.9167 E	off Poulithra Leonidiou, East Peloponnese, Greece	PELAGOS CETACEAN RESEARCH INSTITUTE	897	73
**7**	2020, June 11th	38.5827 N	16.5928 E	Badolato (Golfo Squillace), Calabria, Italy	CESRAM	1980	48
8	2020, June 12th	37.9630 N	16.1108 E	Brancaleone, Calabria, Italy	BLUE CONSERVANCY ETS	620	25
**9**	2020, June 13th	37.7269 N	15.3218 E	Riposto‐Torre Archirafi, Gulf of Catania, Sicily, Italy (5:30 a.m.)	FISHERMEN (MARECAMP)	9673	814
**10**	2020, June 13th	37.5497 N	15.1669 E	Aci Castello‐ Catania, Gulf of Catania, Sicily, Italy (11:00 a.m.)	MARECAMP	1200	283
	2020, June 13th	37 N (est.)	15 E (est.)	S. Tecla‐Acireale‐Catania‐Agnone Bagni, Gulf of Catania, Sicily, Italy	SAILORS, MARECAMP	1800	20
**11**	2020, June 13th	37.3126 N	15.1887 E	Brucoli, Gulf of Catania, Sicily, Italy (15:30)	MARECAMP	2300	45
**12**	2020, June 13th	37.4869 N	15.1025 E	Near the harbour of Catania, Sicily, Italy	MARECAMP	300	20
**13**	2020, June 13th	37.5037 N	15.0971 E	Near the harbour of Catania, Sicily, Italy (7:00 p.m.)	MARECAMP	20	3
**14**	2020, June, 14th	37.9709 N	15.4255 E	Roccalumera, Messina, Sicily, Italy	MARECAMP	2394	320
**15**	2020, June, 14th	38.1166 N	15.5333 E	Mili Marina, Strait of Messina, Sicily, Italy	MARECAMP, MUMA MILAZZO	1340	379
**16**	2020, June, 14th	38.2764 N	15.6610 E	Torre Faro, N of Strait of Messina, Italy	MUMA MILAZZO	1120	213
**17**	2020, June, 20th	42.6783 N (est.)	10.1463 E (est.)	Elba—Pianosa islands, Italy	MARINA DI CAMPO DIVING CENTER	5360	78
**18**	2020, June, 22nd	44.1322 N (est.)	8.2877 E (est.)	1 nm S of Pietra Ligure, Italy	WALTER SPAGNA	1300	76
**19**	2020, June, 23rd	44.3848 N (est.)	8.9927 E (est.)	Genova Quarto, Italy	GOLFO PARADISO WHALE WATCHING	500	30
**20**	2020, June, 23rd	44.4136 N (est.)	8.8019 E (est.)	Genova Pegli, Italy (11:45 a.m.)	GOLFO PARADISO WHALE WATCHING	300	25
**21**	2020, June, 23rd	44.3561 N	8.5966 E	Varazze, Italy	MENKAB, DISTAV‐GENOA UNIVERSITY	482	6
**22**	2020, June, 24th	43.9934 N	8.1675 E	Alassio, Italy	DELFINI DEL PONENTE	200	10
**23**	2020, June, 24th	43.8693 N (est.)	8.0115 E (est.)	Imperia (Porto Maurizio), Italy	TRI	279	20
**24**	2020, June, 25th	43.6792 N	7.3450 E	Cap Ferrat, France	SOS GRAND BLEU	461	134
**25**	2020, June, 26th	43.5803 N (est.)	7.1320 E (est.)	Antibes, France	SAILORS	_	_
**26**	2020, June, 27th	43.2282 N (est.)	6.6657 E (est.)	Pampelonne, Ramatuelle (Gulf of Saint‐Tropez), France (3:30 p.m.)	MIRACETI, PORT‐CROS NATIONAL PARK	250	17
**27**	2020, June, 29th	43.0666 N	6.3833 E	Cap Bénat—The Levant Island, France (10.45 a.m.)	PORT‐CROS NATIONAL PARK, WWF FRANCE	2700	71
**28**	2020, June, 29th	43.0300 N	6.2150 E	Porquerolles, , France	PORT‐CROS NATIONAL PARK/WWF FRANCE	1780	19
**29**	2020, June, 30th	43.0494 N	6.2233 E	N of Cap des Mèdes, Porquerolles, France (9.50 a.m.)	WWF FRANCE, MIRACETI, PORT‐CROS NAT. PARK, DECOUVERT DU VIVANT	2950	24
**30**	2020, July 1st	43.0365 N	6.2418 E	N of Cap des Mèdes, Porquerolles, France	VERTICAL HORIZON	1000	23
**31**	2020, July 2nd	43.0188 N	5.8731 E	Gulf of Toulon, France (11:20 p.m.)	WWF FRANCE, MIRACETI	3210	301

### 2.1 1996–2019

With a peculiar manner of diving, raising her tail well above the surface and showing a unique shape of the left fluke, this whale was easily recognized during many encounters over the years, within or close to the Pelagos Sanctuary. However, the positive identification of the individual also included the matching of blaze, chevron, dorsal fin shape, and a notch before the dorsal fin, which had already been in place since the first sighting in 1996.

Codamozza was biopsied by WWF France, twice in 2006 and once in 2013, by means of a biopsy dart fired from a crossbow, and was determined to be a female (Tardy et al. 2023); two half‐siblings were also identified in the genetic catalog (Céline Tardy, *personal communication*).

### 2.2 2019–2020

After the second accident that completely severed her tail, making her even more unmistakable, Codamozza was sighted in several locations, within and far from the Pelagos Sanctuary (Table 2).

In two instances, images were obtained by means of a drone (in both cases, DJI Mavic 2 Pro). Behavior and body condition were initially evaluated by images taken in Calabria (Italy, June 12, 2020). Further aerial images were obtained between Cogoleto and Savona (Italy, June 23, 2020), with the additional aim of estimating the whale's body length. The measurement in pixels was compared to the length of the adjacent boat and converted to meters, following Christiansen et al. (2016).

The whale's behavior was evaluated by direct observation, pictures, and videos, including underwater footage in Sicily, Italy, on June 13, 2020 (by a Zenolige Action Cam 4K 12MP FHD 1080P 170° Ultra wide angle).

Dive times of flukeless Codamozza were recorded on June 13, 2020 in the Gulf of Catania (by Marecamp Association) and on June 24 in Liguria, first between Laigueglia and Imperia (by Delfini del Ponente APS) and further, on the same day, in front of Imperia (by TRI). These data were pooled together and analyzed considering dives (interval between two breaths spaced by 26 s or more) and surfacing times (sum of consecutive inter‐blow intervals lasting less than 26 s), according to Jahoda et al. (2003).

The healing process of the wound was evaluated through photographs collected between September 2019 and July 2020.

## 3 Results

In the TRI's fin whale photo‐identification catalog, initiated in 1990, no pictures of Codamozza before 1996 were found, nor was she located in other catalogs started in the subsequent years (GREC, Groupe de Recherche sur les Cétacés, EPHE Ecole Pratique des Hautes Etudes, EcoOcéan Institut and Centre d'Études Biologiques de Chizé) (Zanardelli et al. 2022).

**FIGURE 1:**
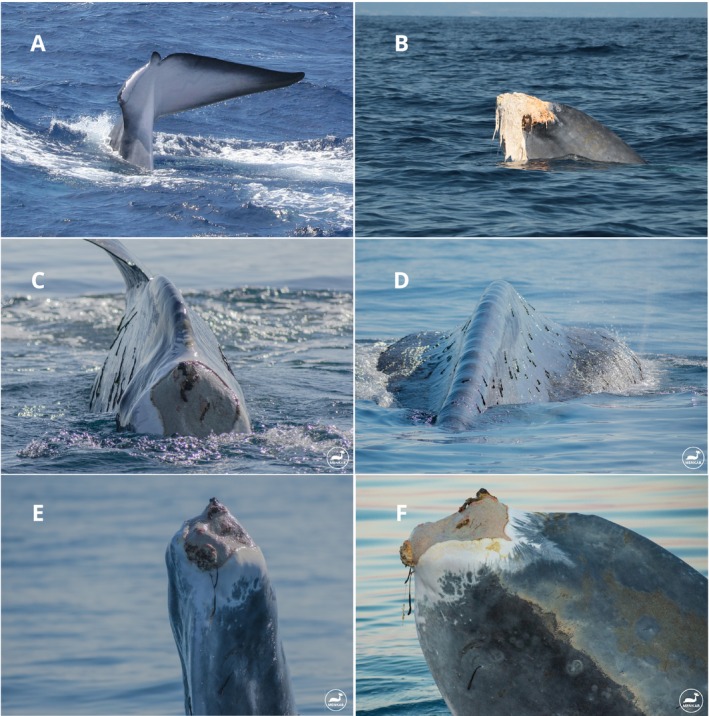
Codamozza (A) August 2005 (photo: C. Lanfredi/Tethys Research Institute). (B) October 2019 (photo: T. Martin/SOS Grand Bleu). (C–F) June 2020 (photo: B. Violi/Menkab: il respiro del mare).

Between 1996 and 2019, that is, prior to her second accident (Figure 1A), we collected 16 observations of Codamozza and, almost certainly, other whale‐watchers and recreational boaters spotted her on several occasions. She appeared to regularly spend her summers in the north‐western part of the Pelagos Sanctuary.

While fin whales usually show only the caudal peduncle, not the flukes, before diving, Codamozza used to raise her tail vertically above the surface, using speed and a porpoising behavior to facilitate her dives. The same behavior was observed in another individual, missing part of its flukes, in 1994 (see list above), and was also described by Urbàn et al. (2004) for a flukeless gray whale.

The silhouette of Codamozza's tail was rather distinctive. The right fluke was intact, with a strikingly white underside and a black margin all around, wider at the tip and on the trailing edge. The left fluke was almost completely amputated with a regular convex curve, leaving only one‐fifth of the surface and a small appendix near the median notch of the tail, pointing laterally. This appendix had a white underside with a black margin, like its right counterpart; but the edge of the cut fluke had healed into a white scar below. At the time of the first sighting, the wound appeared already perfectly healed.

On August 18, 2019, Codamozza was photographed off Île du Lévant (Port‐Cros National Park, France) in good shape, with her partially amputated left fluke. Less than a month later, on September 14, fishermen reported a severely injured whale near Saint‐Jean‐Cap‐Ferrat (France). The same individual was then photographed in the same area on October 5. The distinctive shape of the hump and the notch at the base of the leading edge of the dorsal fin were diagnostic: it was identified as Codamozza, which had suffered a new dramatic accident.

This time, the flukes were totally amputated near the end of the caudal peduncle. Large pieces of necrotic, whitish flesh, over 1 m long, were hanging on each side of the stump (Figure 1B). The skin was missing from the last part of the caudal peduncle, peeled off for approximately 0.7–1 m. Above the wound margins on the flanks and back, the skin appeared lighter, as though it had been scrubbed off by some large object. At least 14 short, roughly parallel scars were visible on the back of the peduncle. In the middle of the back, a large oblong light gray mark, surrounded by dark skin, was running for 1–2 m, also seeming to be the result of a scraping. Despite these wounds, the whale remained capable of swimming and diving.

In the following weeks, Codamozza began to move westward. She was sighted with another fin whale on October 13, 2019, near the northern shore of Île du Lévant, and alone again, near the French–Spanish border, on October 26 and 27, 2019. This was the last sighting of Codamozza for nearly 7 months. No evident emaciation was noticed between August and the end of October 2019.

On May 20, 2020, she was unexpectedly seen and filmed 18 km from Jableh, south of Latakia (Syria). On June 5, after covering at least 1190 km in 16 days, traveling west‐northwest, she was observed close to Leonidio, east of the central Peloponnese (Greece).

The large hanging pieces of flesh had disappeared, and the skinless stump ended with a massive fibromuscular or keloid mass, light gray in color, appearing dense, from which some orange‐red protrusions, possibly tips of vertebrae, extended (Figure 1C,E,F). The skin, which had turned white during the healing process, could not cover the stump and encircled it in an irregular pattern.

**FIGURE 2:**
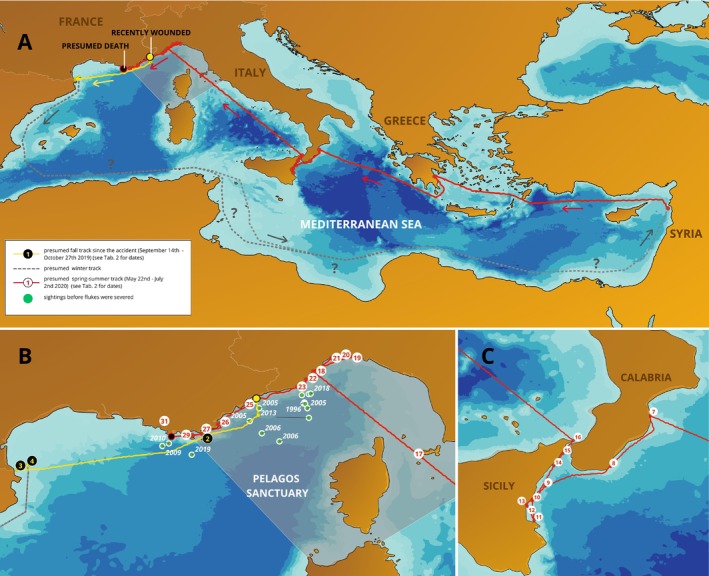
(A) The journey of Codamozza from the approximate point where she was first seen without flukes to the point of presumed death. Lines between sighting points show the shortest route. The segment between the French‐Spanish border and Syria (gray dotted line) is speculative, and two possible routes were hypothesized: One following the coastal currents along Africa; another passing by Lampedusa, which has been described as a winter feeding ground for fin whales (Canese et al. 2006). (B) Sighting points of Codamozza before (green dots) and after becoming flukeless (yellow line, black dots in fall; red line, white dots in spring/summer). (C) Sightings in southern Italy. Numbers refer to sighting points listed in Table 2.

Leaving the eastern Mediterranean basin, the whale headed westward and arrived off the east coast of Calabria (Southern Italy) on June 11 (Figure 2A). In the following days, she first headed south to the Gulf of Catania (Sicily) and then back north, passing twice through the Strait of Messina. She then proceeded north, bound for the Pelagos Sanctuary. She was spotted again between the islands of Elba and Pianosa in the northern Tyrrhenian Sea. Finally, she was seen off the northern shore of the Ligurian Sea, in front of Pietra Ligure on June 22. Here, she slowed down, eventually moving back eastward to Genova and then again toward Celle Ligure and Savona on the West. She then continued her westward journey, swimming close to the coast. She already appeared extremely emaciated (Figure 1D), and after reaching Beaulieu‐sur‐Mer (France), she remained motionless near the shore, 65 m from the coastline, for an entire morning. She then moved eastward up to the Port‐Cros National Park at a slower pace.

At this point, Codamozza was swimming with even greater difficulty and spent the following day motionless in the same area between Hyères and the archipelago in front of it. On July 1, Codamozza swam only 4.5 km, departing from the island of Porquerolles and reentering the Pelagos Sanctuary in the evening. On July 2, she was in the Bay of Toulon, motionless: that was the last sighting of Codamozza.

Western winds arose in the following days. Not surprisingly, no stranding was recorded and no body was found at sea; Codamozza was so emaciated that she had no blubber left and very likely died, sinking to the bottom (Dhermain 2020).

The extreme cachexia of Codamozza, most likely due to the negative caloric balance, was already evident in the waters of the Peloponnese, 1 month before her presumed death. Most of the dorsal muscles and blubber had disappeared, leaving the backbone conspicuously protruding. On her hollow flanks were hanging hundreds of copepods *Pennella balaenopterae* . These ectoparasites are not harmful per se (although they may increase friction) but their number indicates how bad the whale's body condition was. A further slight loss of body mass could be noticed between Catania (June 13) and Port‐Cros (June 26).

The lack of use of her dorsal muscles and/or possible denervation could have induced a loss of muscular mass (Bruno Cozzi, University of Padua, Italy, *personal communication*). Using muscles other than the epaxial and hypaxial dorsal ones could have demanded more energy than usual, contributing to the severe emaciation.

In her last days, in France, the dorsal fin was hanging on the right side, a sign of muscle degradation at the base of the fin, or possibly a consequence of the lack of pressure on each side of the fin, assuming she could not perform deep dives.

Drone and underwater videos showed that the terminal part of her body was devoid of mobility and she swam energetically using her pectoral fins for support, in a kind of breaststroke. This behavior has been witnessed before in humpback whales (Henry et al. 2020) and subsequently in “Mezzacoda,” a similarly injured fin whale (see above). Codamozza was not using an undulation of the back of the body, nor was she swimming on her side moving the peduncle laterally as described in flukeless gray whales (Gilmore 1959; Urbàn et al. 2004).

On a few occasions, Codamozza traveled > 100 km/day. Although a flukeless gray whale was once reported to have completed its annual migration at a rate of 72–80 km/day (Gilmore 1959), the resilience of this disabled fin whale was unexpected. However, her calculated speed appears variable: rather low immediately after the second accident (0.6 km/h) and during the last 9 days before presumed death (1.7 km/h), when the animal was likely suffering and devoid of energy. However, from the time she showed up in Syria in May until she reached the northern coast of the Sanctuary, her calculated speed (4.49 km/h), as well as her measured speed (4 km/h in the Gulf of Catania and 3.28 km/h along the Ligurian coast), were only slightly less than the mean of 1.3 m/s (4.68 km/h) calculated by Jahoda et al. (2003) for healthy Mediterranean fin whales.

Except for the first days after the accident, flukeless Codamozza traveled close to shore (mean distance from the coast 3276 m, mean depth of sighting points 190 m, *N* = 30), with only possible straight crossings between the Peloponnese and Calabria, and between Elba and Liguria. This is in striking contrast to her previous behavior, when, with one fluke still in place, she was typically sighted in the pelagic area, as is common for fin whales in the Sanctuary (Panigada et al. 2005; Azzellino et al. 2008). Sighting points before the loss of her tail were recorded at a mean distance from shore of 33 km and a mean depth of 2248 m (*N* = 16). Both depth and distance from the coast were significantly different before versus after the amputation of her tail (*t* test, *p* < 0.0001).

In some instances, and especially at the end of her journey near Beaulieu‐sur‐Mer (France), the whale was observed spending much time motionless logging at the surface. Her mean surface time (150.1 s, *N* = 55) was significantly longer compared to healthy fin whales in Jahoda et al. (2003) (mean = 90.0 s, *N* = 18) (*t* test, *p* < 0.05). However, Codamozza's mean dive times (175.7 s, *N* = 74) were not significantly different from those of other fin whales (Jahoda et al. 2003) (mean = 227.5 s, *N* = 18) (*t* test, *p* > 0.05). Nonetheless, it should be noted that Codamozza was never observed performing dives longer than 8 min, while fin whales are known to reach up to 17.3 min in the Atlantic (Fonseca et al. 2022).

Aerial images provided a body length measurement (with no tail) of 15.93 m (SD = 0.06). As Codamozza already appeared to be adult when first sighted in 1996, her age at death must have been at least 37 years, considering that female fin whales reach 95% of their asymptotic length at the age of 13 (Aguilar and Lockyer 1987).

## 4 Discussion

### 4.1 Cause of Injuries and Death

It is highly likely that Codamozza died of starvation. However, since her body was not retrieved, no additional lethal factors could be investigated, such as morbillivirus infection, which typically affects weakened animals (Mazzariol et al. 2016), or intoxication by pesticides and heavy metals, that may have been stored in the blubber and released into the bloodstream due to abnormal blubber consumption (Pinzone et al. 2015).

Anthropogenic causes are almost certainly to blame for both the whale's accidents.

When Codamozza was first observed, her partially cut off left fluke was already perfectly healed. Based on the healing process observed between the amputation in August/September 2019 and June 2020, it can be assumed that the first injury occurred at least 9 months before August 1996, when she was first sighted. The curved shape and the regularity of the cut on the left fluke suggest a ship strike, as does the large notch before the dorsal fin, which appears wider than if it had been caused by a rope, suggesting that both injuries may be due to the same accident. However, regarding the notch, we cannot definitively rule out an entanglement, which could have occurred previously and widened over the years.

The cause of the second accident is even less clear. On August 18, 2019, Codamozza still appeared healthy; until then, she had been perfectly able to feed, having been observed for 23 years in unchanged body conditions. Only 27 days later, on September 14, she had lost her flukes. A long‐term consequence of the first amputation can be ruled out. The previous accident did not affect the caudal peduncle, and there was no plausible reason for such necrosis, neither parasitological nor hematological.

The two main hypotheses are a second ship strike, resulting in an immediate amputation of the tail, or an entanglement; both are known causes of flukes' amputation (Moore et al. 2013). However, a second ship strike does not appear to be the most likely. A clean severing of the caudal peduncle by a ship strike would have led to a massive hemorrhage and would likely have been lethal in the short term (Wiley et al. 2016). Although bleeding could have been reduced by the super‐tonus of elastic dermis, muscles, and blubber, cetaceans lack a coagulation factor in order to prevent strokes and diving‐related accidents, and the arteries in the region are quite large (Cozzi et al. 2017; Huggenberger et al. 2018).

Instead, the scars on the caudal part of the back of the whale, between the dorsal fin and the stump, rather point to a friction of ropes or a net. Thus, the second accident may be the result of an entanglement. The whale's strength and velocity could have contributed to driving the ropes into the flesh. Tight wraps at the base of the flukes could have created a ligature that constricted blood flow, contributing to the necrosis of the tail, potentially already partially cut off by the ropes. This could better explain how the loss of the tail occurred without massive and immediate lethal bleeding. However, the rapidity of the whole process raises questions, as such an event occurred in less than 27 days. Although the particularly hypertonic environment in the Mediterranean Sea, where salinity reaches 37‰, may have accelerated the healing, and cetaceans are well known for their ability to recover from severe wounds (Su et al. 2022), this is definitely a very short time.

### 4.2 Migration

Even without her usual means of propulsion, Codamozza was still able to complete a round trip between north‐western and eastern Mediterranean. Assuming the shortest route between Spain and Syria (where no sightings of her were reported), she covered a total distance of over 7000 km in almost 9 months. Had she instead swam along the coast, as she typically did when observed on her westward journey, Codamozza's trip would have been quite longer, approximately 8000 km (Figure 2A).

Movements of fin whales outside their summer feeding grounds in the Pelagos Sanctuary and the adjacent area have only been documented for short periods and for a limited number of individuals (Panigada et al. 2017; Panigada et al. 2024). Codamozza could be tracked for a large part of her migration over a timespan of almost a year, providing new evidence of unknown large‐scale movements of a fin whale within the Mediterranean.

It has been hypothesized that Mediterranean fin whales move as nomadic opportunists rather than following a clear migration pattern along defined routes as is common in other *Mysticetes* (Geijer et al. 2016; Notarbartolo di Sciara et al. 2016). Whether Codamozza's route is representative of typical Mediterranean fin whale movements across the basin, or if her impairment caused her to alter usual behavior, remains a subject of speculation.

In October 2019, Codamozza left the Pelagos Sanctuary and moved westward, a behavior observed in other fin whales at the end of summer (Panigada et al. 2017, 2024). She reappeared in May 2020, rather unexpectedly, in Syria, very close to the easternmost part of the Mediterranean Sea. In the Levantine Basin, sightings of fin whales are quite rare (Notarbartolo et al. 2003; Stephens et al. 2021), suggesting, on one hand, that the disabled Codamozza may have found herself off course. On the other hand, other factors are not in contrast with the hypothesis that her path may have been within the norm. The low number of sightings in the eastern basin may be due to the lack of systematic surveys. Where more regular observations have been conducted, such as in the waters off Israel, several sightings are on record (Kerem et al. 2012). Furthermore, Druon et al. (2012) developed models indicating the existence of potential feeding habitats for fin whales in the Levantine Basin from autumn to spring. In any case, to the best of our knowledge, the sighting of Codamozza off Syria represents the first documented connection between fin whales seen in the Ligurian Sea and the Eastern Basin.

Codamozza may have utilized currents flowing eastward along the coast of Africa, which could have taken her to the extreme eastern limit of the basin, whether it was her intention or not. Subsequently, she traveled all the way up to the Pelagos Sanctuary, staying unusually close to the coast, possibly again taking advantage of surface currents. A similar behavior was observed in 2021 when a similarly emaciated and likely exhausted young gray whale, named “Wally,” which had entered the Mediterranean Sea, followed the same counterclockwise route from the Gulf of Naples to Spain (Dhermain 2024). One further possibility is that Codamozza's path may be the result of a combination of a “normal” route and favorable currents exploited to balance decreasing fitness. In any case, given that migratory patterns of Mediterranean fin whales are still quite uncertain (Geijer et al. 2016), her track adds a further element to the picture.

### 4.3 Public Awareness

Arising from two distinct and most likely human‐induced accidents, Codamozza's case was emblematic from the start, highlighting a type of incident that probably goes unnoticed in numerous instances. After the dramatic amputation of her whole tail, her story garnered substantial attention in both Italian and French media, as well as in non‐Mediterranean countries (Austria, UK). Notably, this also captured the attention of the Italian Ministry of the Environment, prompting its Sea and Coast Protection Directorate to seek the opinions of national experts. On several occasions, the Italian and French Coast Guards assisted in keeping boats at a distance.

Regardless of the cause of the whale's second accident, dates and locations of the sightings close to this event strongly suggest that it occurred within the Pelagos Sanctuary—ironically in the place specifically designated for the protection of marine mammals in the Mediterranean, and an Important Marine Mammal Area (IMMA), as identified in 2016 by the Marine Mammal Protected Areas Task Force of the International Union for Conservation of Nature (IUCN) (Tetley et al. 2022).

As flagship species beloved by the public, cetaceans can elicit strong human responses. Narratives that focus on a single charismatic individual, especially when in distress, often capture more public attention than statistics alone. Through effective communication via mass media, cases like that of Codamozza can generate substantial public support for the conservation and welfare of cetaceans, emphasizing their ecological significance and potentially garnering broader support for the conservation of marine ecosystems as a whole (Reamer et al. 2024).

Drawing the general public's attention to collisions with vessels and entanglement in fishing gear—two of the most serious threats to large cetaceans in the Mediterranean and worldwide—could hopefully provide the basis for effective management of these impacting anthropogenic activities. For instance, with regard to shipping traffic, public support could potentially bring immediate benefits: currently, apart from zones where areas to be avoided can be easily implemented (Frantzis et al. 2019), the most effective and only demonstrated measure to prevent collisions between cetaceans and ships is the reduction of vessel speed (Panigada et al. 2020; Sèbe et al. 2023). Passengers willing to travel at slower speeds in exchange for enhanced protection of marine life could represent a pivotal influence on ferry companies.

## 5 Conclusion

Codamozza's case emphasizes the urgent need for more effective conservation measures, for which additional data on medium‐to‐large scale movements would be needed. It also highlights the significant challenge of protecting a highly mobile species even in a relatively small basin such as the Mediterranean Sea. Above all, it suggests the imperative for a broader, ecosystem‐based approach to complement area‐based measures that are already in place.

In the broader context of conservation, as pointed out by Notarbartolo di Sciara and Würsig (2022), it is not simply a matter of addressing mortality causes or merely maintaining population status if survival means that these animals must constantly struggle against threats such as, but not limited to, entanglement in fishing gear or ship strikes. Rather than solely keeping population levels within a safe zone, the primary goal should be to limit human pressures in the first place.

Last but not least, humans bear a moral obligation to prevent extreme suffering in animals (Moore and van der Hoop 2012), thereby averting dramatic accidents like Codamozza's, probably among the most painful and disabling events that can happen to a whale.

## Author Contributions

**Maddalena Jahoda:** conceptualization (lead), data curation (lead), investigation (equal), visualization (lead), writing – original draft (lead), writing – review and editing (lead). **Margherita Zanardelli:** conceptualization (equal), data curation (equal), investigation (equal), writing – original draft (equal), writing – review and editing (equal). **Frank Dhermain:** conceptualization (equal), data curation (equal), investigation (equal), writing – original draft (equal). **Jessica Alessi:** data curation (supporting), writing – review and editing (supporting). **Filippo Armonio:** data curation (equal). **Marco Ballardini:** data curation (equal). **Alain Barcelo:** data curation (supporting). **Giulia Calogero:** data curation (equal), writing – review and editing (supporting). **Elena Fontanesi:** data curation (equal), writing – review and editing (supporting). **Alexandros Frantzis:** data curation (equal), writing – review and editing (supporting). **Maria Assunta Menniti:** data curation (supporting), investigation (supporting). **Clara Monaco:** data curation (equal), investigation (equal), writing – review and editing (supporting). **Céline Obadia:** data curation (supporting). **Giuliana Pellegrino:** data curation (supporting). **Alessandra Raffa:** data curation (supporting), investigation (supporting). **Céline Tardy:** data curation (equal), writing – review and editing (supporting). **Alessandro Verga:** data curation (equal), investigation (supporting). **Biagio Violi:** data curation (equal), writing – review and editing (supporting). **Simone Panigada:** writing – review and editing (equal).

## Ethics Statement

No approvals were required.

## Conflicts of Interest

The authors declare no conflicts of interest.

## Supporting information


Data S1.

## Data Availability

The data that supports the findings of this study are available in the Supporting Information of this article.
