# Supplementary material for: Codamozza‐Fluker: The Compelling Case of a Flukeless Fin Whale Traveling Throughout the Mediterranean Sea and the Need for Basin‐Wide Conservation Efforts
Source: Ecol Evol. 2025 May 21;15(5):e71313. doi: 10.1002/ece3.71313 (PMC12094965; doi:10.1002/ece3.71313)
Supplement: Supplementary file 1 — Data S1. [file ECE3-15-e71313-s001.zip › ece371313-sup-0003-Videos Codamozza (links).docx]

LINKS Codamozza

Please find:

A video by Menkab, showing the very emaciated whale in the Ligurian Sea

<https://vimeo.com/433098380>

A video by Blue Conservancy also showing the whale underwater using her pectoral fins for swimming.

<https://www.youtube.com/watch?v=mxqslemqz1s>
