# Supplementary material for: Codamozza‐Fluker: The Compelling Case of a Flukeless Fin Whale Traveling Throughout the Mediterranean Sea and the Need for Basin‐Wide Conservation Efforts
Source: Ecol Evol. 2025 May 21;15(5):e71313. doi: 10.1002/ece3.71313 (PMC12094965; doi:10.1002/ece3.71313)
Supplement: Supplementary file 1 — Data S1. [file ECE3-15-e71313-s001.zip › ece371313-sup-0002-pictures supplementary info codamozza.pdf]

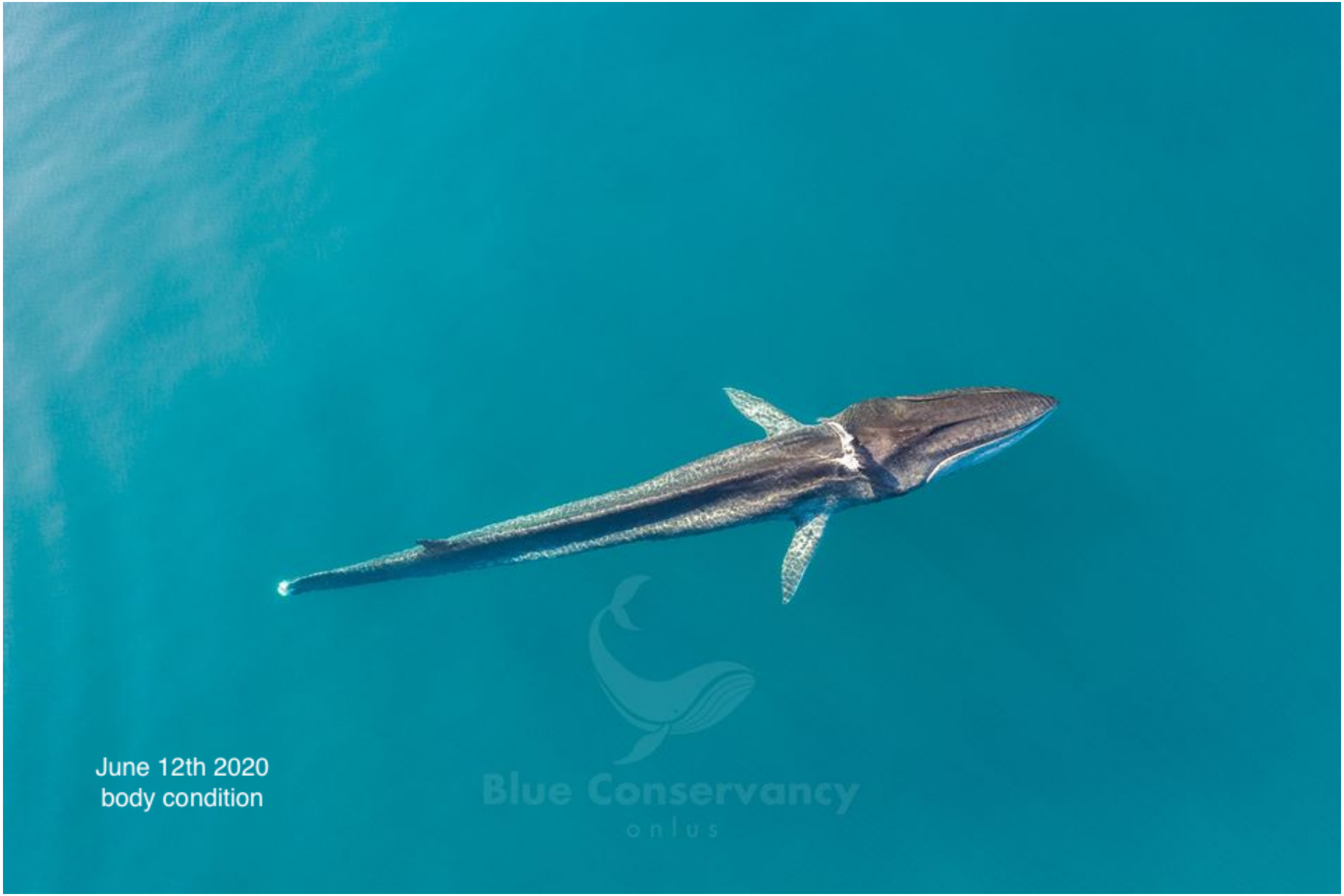
June 12th 2020
body condition
Blue Conservancy
onlus

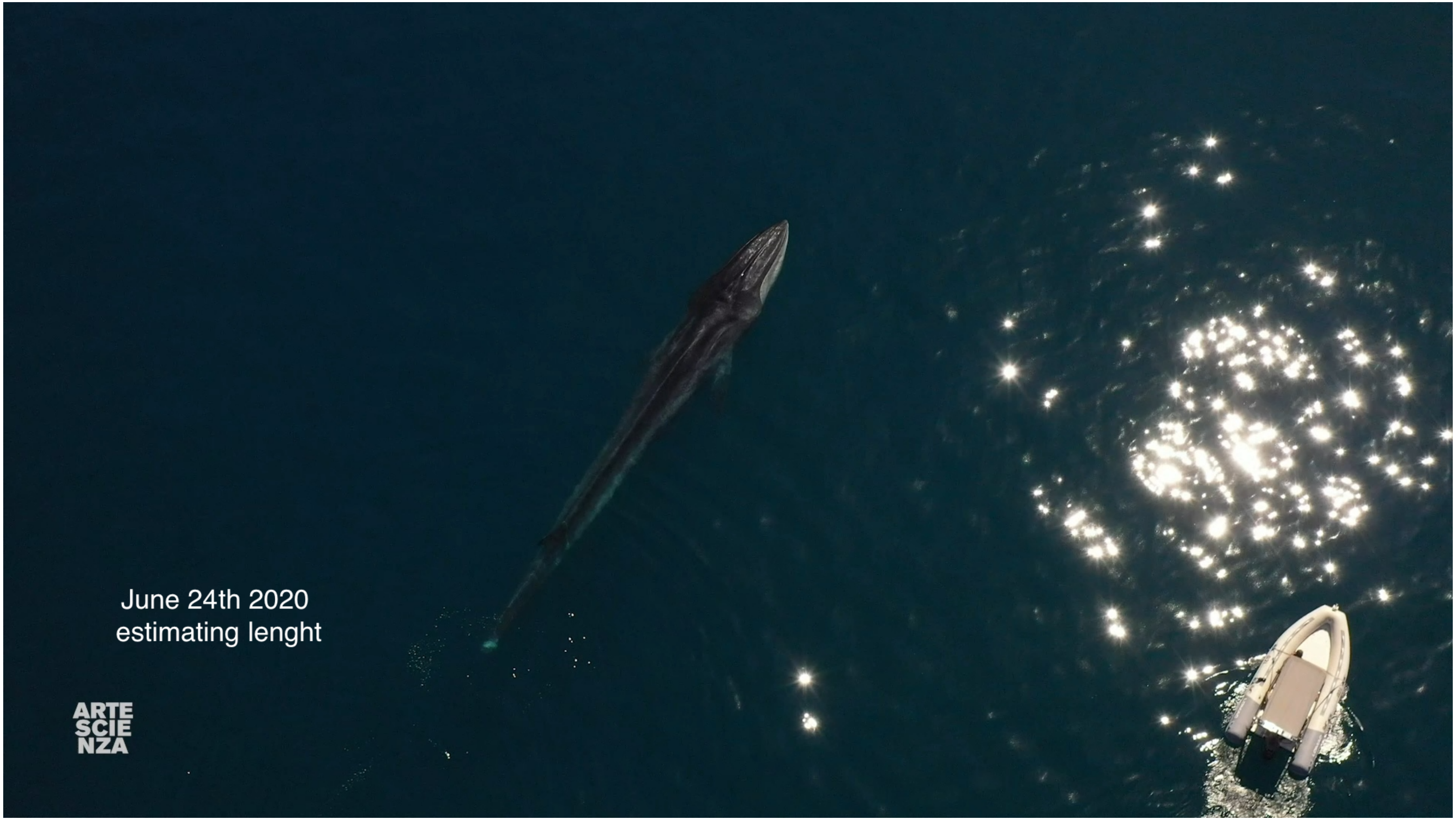
June 24th 2020
estimating lenght
ARTE
SCIE
NZA

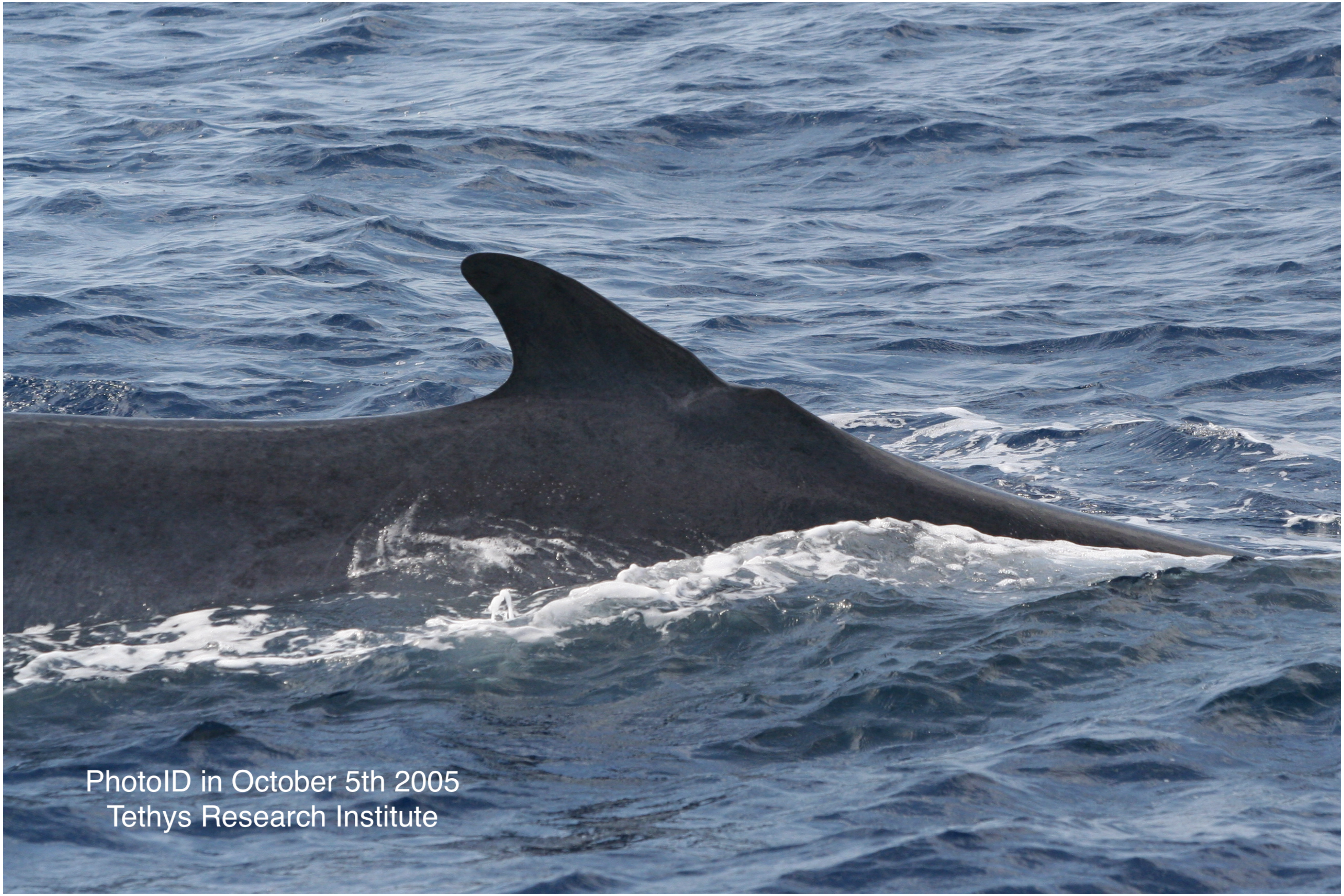
PhotoID in October 5th 2005
Tethys Research Institute

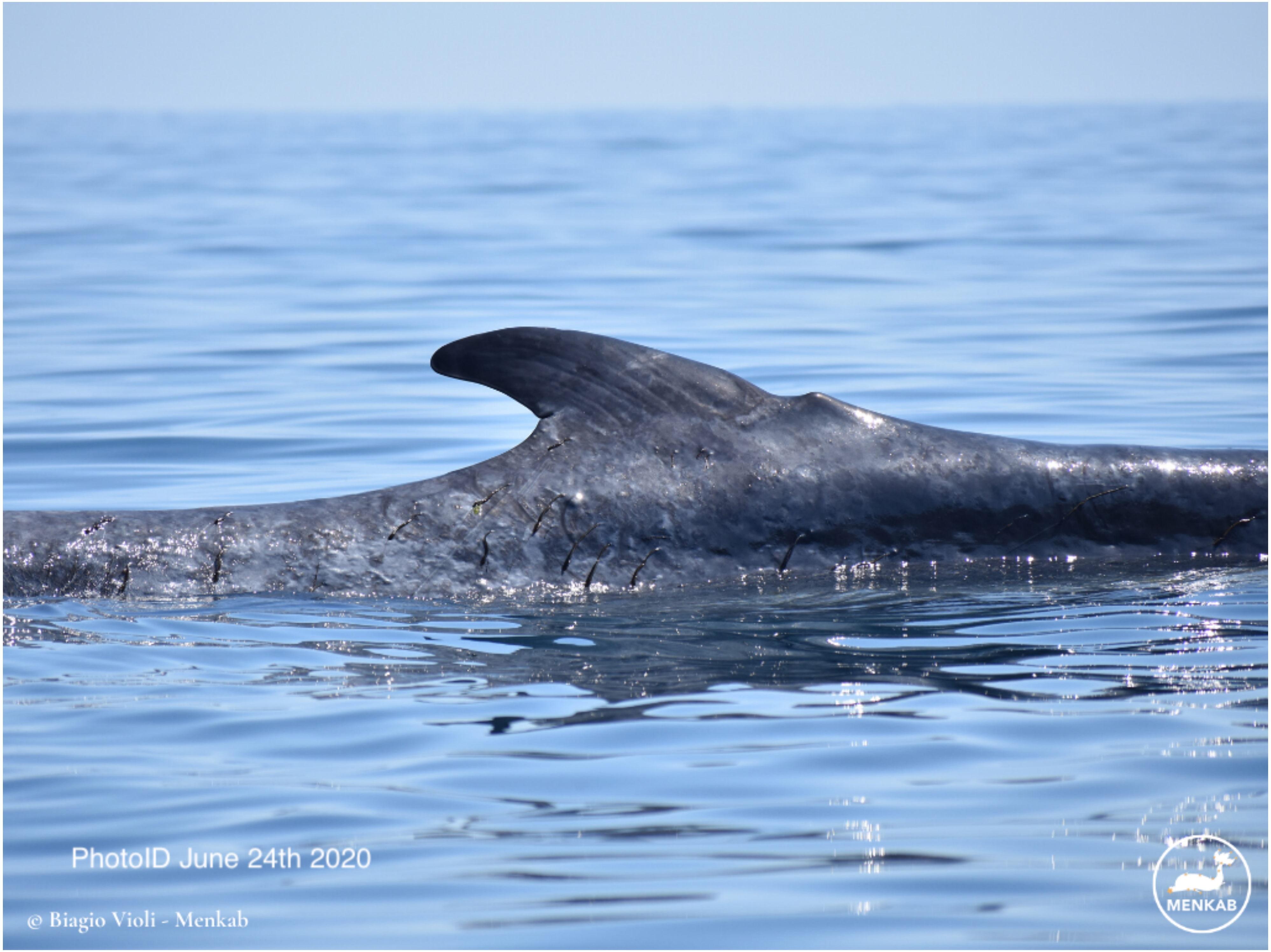
PhotoID June 24th 2020
© Biagio Violi - Menkab
MENKAB
